# Editorial: Machine Learning and Decision Support in Stroke

**DOI:** 10.3389/fneur.2020.00486

**Published:** 2020-05-29

**Authors:** David S. Liebeskind, Fabien Scalzo

**Affiliations:** Neurovascular Imaging Research Core and UCLA Stroke Center, Department of Neurology, University of California, Los Angeles, Los Angeles, CA, United States

**Keywords:** stroke, imaging, machine learning, ischemia, perfusion

Real-world, intelligent application of artificial intelligence (AI) for augmented stroke care has dramatically expanded in the last several years. In this volume, we use the term AI to refer to its popularized definition, more precisely referred to as machine learning (ML) and related to the automatic learning of a computational model from a previously acquired labeled dataset. The use of ML for decision support in stroke has been adopted as a key research priority in most academic institutions while industry partners have flourished, regulatory agencies have adapted to keep pace and clinicians across the globe look to implement these tools to improve stroke care. The application of AI and its combination with imaging has the potential to transform how stroke care is delivered worldwide. Over the last decade, AI has attained several technology milestones by capitalizing on big data, providing mobile solutions, relying on cloud computing, implementing full automation, and advanced graphical interfaces for better visualization. AI has been a priority investment area for healthcare providers as it is expected to increase productivity, efficiency, and geographical reach of healthcare delivery. AI may also improve the experience of stroke care providers, accelerating decision support with imaging and enabling them to spend more time in direct patient care. Exuberance and seemingly miraculous applications of AI abound in stroke, from detecting the earliest signs of ischemia on CT to delivering key metrics on perfusion or blood flow in specific areas of the brain. This clinical perspective may be quite different from the application of AI in radiology or pathology subspecialties, where potential existential threats abound. At present, most advanced imaging software modules in stroke have revolutionized the use of imaging data, yet AI is often only used in extremely limited aspects. In fact, the use of AI in imaging has been balanced with applications to rapidly glean essential information from electronic health records. The numerous AI methods in this volume reflect predominantly academic collaborations that mirror the continually expanding list of novel software products available on the market. Rapid FDA clearance has facilitated numerous commercial products in recent years, but expansive marketing claims may have subsequently overemphasized their impact in saving lives due to stroke. Unfortunately, there have been gaps in rigorous validation and post-marketing surveillance, with prime emphasis on the ability to modernize and simplify clinical decision making.

The broad array of topics in this volume speak to innovation by content experts who understand far beyond the simple or isolated imaging data. Bang et al. provide their perspective on the development of multimodal MRI triage strategies, including obstacles and achievements along the way. Kamal et al. provide a survey of various ML applications in imaging of acute ischemic stroke. Novel methods to predict CT perfusion lesion growth are offered by Lucas et al.. Almost a dozen more original research articles describe unique ways to apply AI, using innovative methods on CT, MRI, and TCD in a wide variety of settings from around the world to potentially streamline stroke care by advancing decision support (Chan et al.; Dhar et al.; Habegger et al.; Park et al.; Pinto et al.; Thorpe et al.; Ulas et al.; van Os et al.; Winzeck et al.; Wu et al. Xiao et al.). These reports chronicle the remarkable progress achieved in AI for stroke imaging, reflecting an early sea change preceding the expected tsunami of AI stroke imaging tools to flood bedside encounters in coming years.

Gaps undoubtedly remain as AI methods represent only a fraction of these modern tools. Automation of image processing is extremely valuable, but the ML component remains limited. Most AI tools for stroke imaging rapidly generate an existing variable commonly used, such as an ASPECTS score or ischemic core lesion volume. However, the methods, definitions, and ultimate reliability often remain remiss or ill-defined. In such scenarios, there is not much “machine” learning or deep learning, while clinicians will not learn unless they contrast automated results with their own review of the images. Radiology worklists may be quickly sorted to facilitate their priority reads, but sensitivity and specific of these methods are both critical. From the bedside, some clinicians have argued that an “eyeball” method to rapidly glance at the imaging to recognize simple patterns may be enough. The clinical context is therefore essential. For example, standard definitions of core and penumbra on CT perfusion generated by automated software have been developed and validated predominantly in acute, complete occlusion of a proximal artery such as the MCA. Such defined metrics for the delayed perfusion of penumbra or decreased cerebral blood flow are not applicable in more subacute strokes, in the presence of stenoses or in other territories where the collateral flow patterns are different. The exact volume of such lesions is also extremely simplistic, as the topology or pattern is essential information that is poorly captured by machine, yet readily seen by expert eyes. The utility of imaging in stroke for decision support has always been driven by the most subtle findings and focused around the clinical context. For instance, recognizing FLAIR vascular hyperintensities in the distal MCA territory may be critical information for the patient presenting with transient or mild hemi-neglect due to right hemispheric ischemia. Unfortunately, machine learning must be trained by such rich data that incorporates these critical clinical contextual data. Similarly, the development of AI tools should be prompted to address the most pertinent clinical questions in decision support. Clinician input and continual development in light of this perspective is therefore key. The papers in this volume largely reflect this perspective from the bedside application in real-world scenarios. Validation of nascent tools also requires human, clinical expertise to link results with the underlying pathophysiology. As a result, AI stroke imaging methods will inevitably retain dependence on over-reading by experts and they cannot replicate the role of core lab, detailed adjudications.

The future of AI or ML in decision support with imaging is undoubtedly a key facet of modernization in the delivery of stroke care. Modernization, however, does not equate with a complete replacement of current practice or the role of expertise. Electricity was discovered and harnessed to modernize many aspects of daily life after billions of years of electromagnetic energy on our planet. Similarly, AI of stroke imaging will require much clinical expertise to continually modernize stroke care.

## Author Contributions

DL and FS contributed to the creation of this article, from initial drafts to critical revisions, and final production.

## Conflict of Interest

The authors declare that the research was conducted in the absence of any commercial or financial relationships that could be construed as a potential conflict of interest.

